# Death distress constructs: A preliminary empirical examination of the Farsi form in nurses: A brief note

**DOI:** 10.1002/nop2.484

**Published:** 2020-03-23

**Authors:** Mahboubeh Dadfar, David Lester

**Affiliations:** ^1^ School of Behavioral Sciences and Mental Health‐Tehran Institute of Psychiatry International Campus School of Public Health, Student Committee of Education and Development Center (EDC) Spiritual Health Research Center Iran University of Medical Sciences Tehran Iran; ^2^ Stockton University Galloway NJ USA

**Keywords:** death anxiety, death depression, death distress, death obsession, Iran, nurses

## Abstract

**Aim:**

Death distress can increase mental health problems. The aim of the present study was to develop a measure of death distress and evaluate the reliability of this Death Distress Scale‐Farsi (DDS‐F) among nurses. The hypotheses were that death distress has three components and that the DDS‐F would have desirable psychometric properties.

**Design:**

A descriptive cross‐sectional study.

**Methods:**

A convenience sample of 106 Iranian nurses from two hospitals at Tehran city, Iran was recruited. They completed the Death Anxiety Scale (DAS), the Death Depression Scale (DDS) and the Death Obsession Scale (DOS).

**Results:**

Cronbach's *α* for the DDS‐F was 0.71. As expected, the DDS‐F had three independent components: death obsession, death depression and death anxiety. A principle component analysis with a varimax rotation of the DDS‐F items identified three factors accounting for 66.13% of the variance. Factor 1 was labelled “Death Obsession” (31.3% of the variance), Factor 2 was labelled “Death Depression” (21.9% of the variance), and Factor 3 was labelled “Death Anxiety” (12.8% of the variance).

**Discussion:**

Death distress has three components: death obsession, death depression and death anxiety. The DDS‐F which measures these has good psychometric properties, and it can be used in hospital settings to assess death distress among Iranian nurses.

## 1 BACKGROUND

There is a negative attitude towards issues related to death and dying in nurses working in hospitals of Iran (e.g. Arab, Seyed Bagheri, Sayadi, & Heydarpour, 2019; Dadfar & Lester, 2014a; Dadfar, Asgharnejad Farid, Atef Vahid, Lester, & Birashk, 2014; Dadfar, Lester, Asgharnejad Farid, Atef Vahid, & Birashk, 2014; Sharif Nia, Lehto, Ebadi, & Peyrovi, 2016). The death of patients in hospitals is traumatic for hospital staff since the deaths of their patients lead the staff to think that they have failed in their duty.

However, death education has been shown to be effective in changing the negative attitude towards death in nurses and other hospital staff (Dadfar, Asgharnejad Farid, Lester, Atef Vahid, & Birashk, 2016; Dadfar & Lester, 2014b, 2020; Dadfar, Lester, Asgharnejad Farid, Atef Vahid, & Birashk, 2017; Dadfar, Lester, Atef Vahid, Asgharnejad Farid, & Birashk, 2015; Dadfar, Lester, Birashk, Asgharnejad Farid, & Atef Vahid, 2016; Iverach, Ross, Menzies, & Menzies, 2014; Macedo, 2019; McClatchey & King, 2015; Menzies, Zuccala, Sharpe, & Dar‐Nimrod, 2018; Peters et al., 2013).

Nurses who care for dying patients may experience death distress. Various factors can impact on the level of death distress in nurses (Dadfar & Lester, 2020). One of correlates of death distress is cultural religious spiritual issues (Jong et al., 2018). For example, some findings found among English sample (Maltby & Day, 2000a), Muslim Lebanese (Abdel‐Khalek, 1998a), Saudi Arabia sample (Almostadi, 2012) and Iranians (Dadfar, Bahrami, Sheybani Noghabi, & Askari, 2016; Dadfar & Lester, 2017a; Mohammadzadeh, 2015; Mohammadzadeh & Najafi, 2010, 2018).

To screen nurses and other hospital staff for their sensitivity to death and dying and to evaluate death education programmes for hospital staff, it would be useful to have a brief screening instrument to assess their sensitivity to death and dying. Many scales to measure attitudes towards death have been developed, and previous research has indicated that there are three major components: death anxiety, death depression and death obsession. To administer all three Death Anxiety Scale (DAS), Death Depression Scale (DDS), and Death Obsession Scale (DOS) involved a total of 47 items and the goal of the present study was to develop a brief measure of these three components using a brief 9‐item scale.

## 2 PROBLEM IDENTIFICATION

The aim of the present study was to devise a Death Distress Scale‐Farsi (DDS‐F) for nurses to measure three components of death distress: anxiety, depression and obsessive thoughts.

## 3 METHODS

A convenience sample of 106 Iranian volunteer nurses was selected from different wards of two hospitals in Tehran, Iran: Hazrat‐e Rasool General Hospital affiliated with Iran University of Medical Sciences and the Khatom‐Al‐Anbia General Hospital. Inclusion criteria were as follows: nurses working in the wards and an educational level of bachelor's degree or higher. Exclusion criteria were as follows: having medical diseases and mental disorders and receiving individual or group psychoeducational or psychological interventions. The nurses’ participation in the study was voluntary, and anonymity was ensured. The objective of study was explained to the nurses.

### 3.1 Measures

#### 3.1.1 The Death Anxiety Scale

Death Anxiety Scale (DAS; Templer., 1970) has 15 items. It is responded to on a True/False format. The DAS has six reversed scored items (2, 3, 5, 6, 7 and 15). Total scores can range from 0–15. Higher scores indicate more death anxiety. Moderate to high reliability and validity has been reported for the DAS (see Abdel‐Khalek, Beshai, & Templer, 1993; Conte, Weiner, & Plutchik, 1982; Dadfar, Lester, & Abdel‐Khalek, 2018; Dadfar, Lester, Abdel‐Khalek, & Ron, 2018; Durlak, 1982; Gilliland & Templer, 1986; Lester & Castromayor, 1993; Rajabi & Bahrani, 2001; Saggino & Kline, 1996; Soleimani et al., 2016; Soleimani, Pahlevan Sharif, Yaghoobzadeh, Allen, Pahlevan Sharif, Yaghoobzadeh, Allen, & Sharif Nia, 2017; Soleimani, Yaghoobzadeh, Bahrami, Pahlevan Sharif, & Sharif Nia, 2016; Tavakoli & Ahmadzadeh, 2011; Templer, 1970; Tomás‐Sábado & Gómez‐Benito, 2002; Vargo, 1980; Warren & Chopra, 1979).

#### 3.1.2 The Death Depression Scale

Death Depression Scale (DDS; Templer, Lavoie, Chalgujian, & Thomas‐Dobson, 1990). The DDS has 17 items. Two items (11 and 12) control for an acquiescence response set. The DDS is responded to on two True/False and a five‐point Likert formats. In the present study, a 17‐items True/False format was used. Total scores can range from 0–17. Higher scores indicate more death depression. High reliability and validity has been reported for the DDS (see Abdel‐Khalek, Dadfar, & Lester, in submission; Aghazadeh, Mohammadzadeh, & Rezaie, 2014; Dadfar & Lester, 2017b; Dadfar & Lester, 2020; Dadfar & Lester, in press; Mohammadzadeh, Rezaei, & Aghazadeh, 2016; Rajabi, Begdeli, & Naderi, 2015; Sharif Nia et al., 2017; Templer, Lavoie, Chalgujian, & Thomas‐Dobson, 1990; Templer et al., 2002; Tomas–Sabado, Limonero, Templer, & Gómez‐Benito, 2005).

#### 3.1.3 The Death Obsession Scale

Death Obsession Scale (DOS; Abdel‐Khalek, 1998). The DOS has 15 items. It is responded to on a five‐point Likert‐type rating scale ranging No (a), A little (b), A fair amount (c), Much (d) and Very much (e). Total scores can range from 15–75. High reliability and validity has been reported for the DOS (see Abdel‐Khalek, 1998, 2000; Abdel‐Khalek, Al‐Arja, & Abdalla, 2006; Abdel‐Khalek & Lester, 2003; Dadfar, Abdel‐Khalek, & Lester, 2018; Maltby & Day, 2000b; Mohammadzadeh, Asgharnejad Farid, & Ashouri, 2009; Moripe & Mashegoane, 2013; Rajabi, 2009; Tomas‐Sabado & Gomez‐Benito, 2003).

### 3.2 Data analysis

For determination of the normality of the data and equality of variances, the Kolmogorov–Smirnov test and Levene's test were used, respectively. The data were analysed with descriptive statistics (mean, standard deviations) and a principal component factor analysis to identify the number of factors to be retained. The criterion of eigenvalues greater than or equal to 1.0 was followed, and the varimax orthogonal rotation of axes was adopted. The SPSS/WIN 26.0 program was used.

## 4 FINDINGS

Two‐thirds (67%) of the sample were 30–49 years old; 95.3% were women.; 60.4% had a contractual appointment; 67% had work experience of ≥5 years; 87.7% were staff nurses; 78.3% had rotational work shifts; 50.9% had 0–9 patients per shift; and 58% had 0–6 care of end‐stage patients in the past 3 month. 29.9% had participation in resuscitation operations in the past 3 month ≥5 year.

The mean total score on the DDS‐F was 9.62 ± 3.72. Cronbach's *α* was 0.71 for the DDS‐F, denoting high internal consistency. Cronbach's alphas were 0.55, 0.68 and 0.88 for the three DAS, DDS and DOS subscales, respectively.

The criteria for the factor analysis were evaluated using the Kaiser–Meyer–Olkin Measure of Sampling Adequacy (KMO) and the Bartlett Test of Sphericity. The KMO was 0.688, indicating the adequacy of the present sample. Bartlett's Test of Sphericity was 296.051 (*df* = 36, *p* < .001), indicating that the factor analysis was justified for the present sample. Factor analyses (principal component extraction with a varimax rotation) of the items from each of the three scale (DAS, DDS and DOS) were carried out and revealed 3–4 factors with eigenvalues greater than one. Three items were chosen from each scale using three criteria: (a) a high loading (>0.50) on the first factor identified, (b) the items loaded on only one factor and (c) an examination of the content of the items. As a results 3 items were chosen from each scale (a total of 9 items) for the DDS‐F scale.

These 9 items were gain subjected to the same factor analysis (a principal component extraction with a varimax rotation). Three factors were identified (accounting for 66.13% of the variance), confirming the structure of the DDS‐F. Factor 1 (3 items) accounted for 31.3% of the observed variance and was labelled “Death Obsession.” Factor 2 (3 items) accounted for 21.9% of the observed variance and was labelled “Death Depression.” Factor 3 (3 items) accounted for 12.8% of the observed variance and was labelled “Death Anxiety.” There was no item which loaded on two factors (Table 1).

**Table 1:** Factor loadings (≥0.50) of the Death Distress Scale‐Farsi version (DDS‐F) in Iranian nurses

Death Distress Scale‐Farsi version (DDS‐F) Items	Component		
	1	2	3
1. I am not at all afraid to die. (DAS 5)	0.12	0.14	**0.80**
2. The thought of death never bothers me. (DAS 7)	0.03	0.01	**0.80**
3. I feel that the future holds nothing for me to fear. (DAS 15)	−0.13	0.29	**0.52**
4. Hearing the word death makes me sad. (DDS 2)	0.14	**0.78**	0.09
5. Passing by cemeteries makes me sad. (DDS 3)	0.07	**0.74**	0.16
6. I feel sad when I dream of death. (DDS 17)	0.05	**0.75**	0.10
7. I can't get the notion of death out of my mind. (DOS 3)	**0.88**	0.02	0.01
8. I am preoccupied by thoughts of death. (DOS 4)	**0.91**	0.14	0.01
9. I find it greatly difficult to get rid of my thoughts about death. (DOS 5)	**0.91**	0.12	0.03
Eigen value	2.82	1.96	1.16
% of variance	31.3	21.94	12.85
% of total variance	66.13		

Note

Items of high loadings (> 0.50) are given in bold to more clearly differentiate the factors.

Factor 1 (Items 1, 2 and 3): Death Anxiety.

Factor 2 (Items 4, 5 and 6): Death Depression.

Factor 3 (Items 7, 8 and 9): Death Obsession.

## 5 DISCUSSION

The purpose of the present study was to develop a brief screening instrument for death distress to be used for screening nurses and for evaluation death education programs designed to reduce death distress in nurses and other hospital staff. A 9‐item scale was developed to measure death anxiety, death depression and death obsession. Three components were identified for death distress by previous researchers (see Mohammadzadeh, Ashouri, Vahedi, & Asgharipour, 2018). The study showed Cronbach's *α* was 0.71 for the DDS‐F as a whole. Researchers can use the total score for the DDS‐F or, if they wish, measure each component of death distress separately.

Also, some studies have been found significant associations between different constructs of death distress: death anxiety, death depression and death obsession (see Abdel‐Khalek, 2004a, 2004b, 2012; Al‐Sabwah & Abdel‐Khalek, 2006; Alvarado, Templer, Bresler, & Thomas‐Dobson, 1993; Ayyad, 2013; Dadfar, Abdel‐Khalek, & Lester, 2017; Dadfar, Abdel‐Khalek, Lester, & Atef Vahid, 2017; Dadfar & Bahrami, 2016; Dadfar & Lester, 2015, 2016, 2018; Dadfar, Lester, & Bahrami, 2016; Groebe et al., 2018; Lester, 2003; Shiekhy, Issazadegan, Basharpour, & Maroei Millan, 2013; Tomas‐Sabado & Gomez‐Benito, 2004, 2005; Tomas‐Sabado & Limonero, 2007; Zuccala, Menzies, Hunt, & Abbott, 2019).

For obsession and depression, it is suggested to cut some of items from the DOS and the DDS, because some of them seem more to do with anxiety. The best three items were chosen from the first factor of each of the factor analyses, taking into account content. (e.g. we eliminated the loneliness item 4 from the DDS). Overall, the DAS is not a good scale for the measurement of a death anxiety component of death distress. Maybe for death fear/anxiety, we have chosen the items by content rather than what clusters on the factor analysis since they do not cluster on the factor analysis. Therefore, additional to the DAS, DDS, and DOS, using the Collett‐Lester Fear of Death Scale (CLDFS; Collett & Lester, 1969), which has four Your own death, Your own dying, Death of others and Dying of others subscales, is recommended for future studies.

## 6 CONCLUSION

The DDS‐F was developed to assess death distress with its three components (death anxiety, death depression and death obsession). It has good psychometric properties, and it may be useful in hospital settings to assess death distress among Iranian nurses and other staff and to evaluate death education programmes.

## CONFLICT OF INTEREST

The authors declare that there is no funding for the study, and they have no conflict of interest regarding the publication of this paper.

## AUTHOR CONTRIBUTIONS

All authors have agreed on the final version and meet at least one of the following criteria [recommended by the ICMJE (http://www.icmje.org/recommendations/)]: substantial contributions to conception and design, acquisition of data or analysis and interpretation of data; and drafting the article or revising it critically for important intellectual content.

## ETHICS APPROVAL

This paper is based on a doctoral thesis in clinical psychology by the senior author. The Research Ethics Committee of Iran University of Medical Sciences approved this study.
